# Curative-Intent Aggressive Treatment Improves Survival in Elderly Patients With Locally Advanced Head and Neck Squamous Cell Carcinoma and High Comorbidity Index

**DOI:** 10.1097/MD.0000000000003268

**Published:** 2016-04-08

**Authors:** Jin-Hua Chen, Yu-Chun Yen, Hsuan-Chia Yang, Shing-Hwa Liu, Sheng-Po Yuan, Li-Li Wu, Fei-Peng Lee, Kuan-Chou Lin, Ming-Tang Lai, Chia-Che Wu, Tsung-Ming Chen, Chia-Lun Chang, Jyh-Ming Chow, Yi-Fang Ding, Szu-Yuan Wu

**Affiliations:** From the Biostatistics Center and School of Public Health, Taipei Medical University (J-HC, Y-CY); Institute of Toxicology, College of Medicine, National Taiwan University (S-HL, S-YW); Department of Otorhinolaryngology (S-PY, F-PL, M-TL, C-CW, T-MC, Y-FD); Department of Oral and Maxillofacial Surgery (K-CL); Department of Hemato-Oncology (C-LC, J-MC); Department of Radiation Oncology, Wan Fang Hospital (S-YW); Department of Internal Medicine (J-MC, S-YW), School of Medicine, College of Medicine, Taipei Medical University, Taipei; Department of Biotechnology (S-YW), Hungkuang University, Taichung; Department of Ophthalmology, Buddhist Tzu Chi General Hospital (LLW); Department of Otorhinolaryngology, Taipei Medical University-Shuang Ho Hospital (F-PL), Taipei, Taiwan; and Institute of Biomedical Informatics (H-CY), National Yang Ming University, Taiwan.

## Abstract

For locally advanced head and neck squamous cell carcinoma (HNSCC), therapeutic decisions depend on comorbidity or age. We estimated the treatment outcomes of patients with different Charlson comorbidity index (CCI) scores and ages to determine whether aggressive treatment improves survival.

Data from the Taiwan National Health Insurance and cancer registry databases were analyzed, and we included >20-year-old patients with American Joint Committee on Cancer (AJCC) stage III or IV HNSCC (International Classification of Diseases, Ninth Revision, Clinical Modification codes 140.0–148.9) undergoing surgery, chemotherapy (CT), radiotherapy (RT), concurrent chemoradiotherapy (CCRT), sequential CT and RT, or surgery with adjuvant treatment. The exclusion criteria were a past cancer history, distant metastasis, AJCC stage I or II, missing sex data, an age < 20 years, nasopharyngeal cancer, in situ carcinoma, sarcoma, and HNSCC recurrence. The index date was the date of first HNSCC diagnosis, and comorbidities were scored using the CCI. The enrolled patients were categorized into Group 1 (curative-intent aggressive treatments) and Group 2 (best supportive care or palliative treatments).

We enrolled 21,174 stage III or IV HNSCC patients without distant metastasis (median follow-up, 3.25 years). Groups 1 and 2 comprised 18,584 and 2232 patients, respectively. After adjustment for age, sex, and clinical stage, adjusted hazard ratios (95% confidence intervals) of overall death in Group 1 were 0.33 (0.31–0.35), 0.34 (0.31–0.36), and 0.37 (0.28–0.49), and those of all-cause death among patients undergoing curative surgical aggressive treatments were 1.13 (0.82–1.55), 0.67 (0.62–0.73), and 0.49 (0.46–0.53) for CCI scores of ≥10, 5 to 9, and <5, respectively.

Aggressive treatments improve survival in elderly (≥65 years) and critically ill HNSCC patients. Curative nonsurgical aggressive treatments including definitive RT or CCRT might be suitable for HNSCC patients with CCI scores ≥10.

## Abstract 2

Supplemental Digital Content is available in the text

## INTRODUCTION

Curative-intent aggressive treatments for locoregionally advanced (stage III or IV) head and neck squamous cell carcinoma (HNSCC) including combined modality approaches (surgery, radiotherapy [RT], and/or chemotherapy [CT]) are generally required to optimize the likelihood of long-term control^1,2^ because HNSCC is associated with a high risk of local recurrence and distant metastases. These aggressive combined modality approaches include primary surgery, followed by either postoperative RT or concurrent chemoradiotherapy (CCRT),^3–5^ induction CT (CT before surgery and/or RT),^6,7^ CCRT without surgery, and sequential therapy (induction CT followed by CCRT) without surgery. Decisions regarding the optimal sequence and selection of surgery, RT, and CT are made by a multidisciplinary team, which considers key factors such as the primary tumor site, disease extent, individual patient factors (age, comorbidity, and preferences regarding treatment type), and the likely functional consequences and morbidity of each approach. Curative aggressive treatment may not be effective in elderly patients with HNSCC.^8^ For example, the benefits of CCRT may not be noted in elderly patients, and substituting CCRT for altered fractionation may be similarly detrimental to critically ill or elderly patients.^1,8,9^

In Taiwan, comorbidities are common among patients with HNSCC because its etiology primarily includes smoking, alcohol use, and betel nut chewing.^10^ The Charlson comorbidity index (CCI) is a scoring system in which medical illnesses accompanying cancer (i.e., comorbidities) are weighted according to the mortality risk.^11^ The effects of comorbidities on the outcomes of patients with HNSCC have been extensively established.^12^ The CCI is a valid prognostic indicator in patients with HNSCC; it independently predicts survival when used to score comorbidities and is easy to use and readily applicable.^12^ Paleri and Wight demonstrated the applicability of the CCI in comorbidity assessment in a cohort of patients with HNSCC from the United Kingdom:^13^ comorbidities were significantly associated with overall survival but not cancer-specific survival.^14,15^

For locally advanced HNSCC (stage III or IV), the therapeutic decision always depends on the comorbidity or age. Palliative treatments or best supportive care (BSC) may be selected because of multiple comorbidities or old age. However, the benefit of curative-intent aggressive treatments and the optimal therapeutic decisions for elderly patients with locally advanced HNSCC and those with multiple comorbidities remain unclear. In this study, we explored the treatment outcomes of a national cohort to determine whether aggressive treatment improves survival in patients with different CCI scores and ages. Furthermore, we compared survival rates in HNSCC patients with multiple comorbidities who underwent curative surgery or nonsurgical intervention.

## PATIENTS AND METHODS

In this study, data from the Taiwan National Health Insurance (NHI) and cancer registry databases—both covering ∼99% of the entire population of Taiwan—were analyzed to create 2 cohorts. Patients diagnosed with HNSCC from January 1, 2002, to December 31, 2011, were enrolled. The follow-up duration was from the index date to December 31, 2013. The Taiwan NHI Administration releases research-oriented data sets through the Collaboration Center of Health Information Application (CCHIA); these data sets include all original claims data and registration files of beneficiaries enrolled in the NHI program. Thus, researchers can use the CCHIA data for tracing all medical services used for all patients with HNSCC in Taiwan. The cancer registry database of the CCHIA contains abundant cancer-related information, including the clinical stage, treatment modalities, pathology, RT doses, and regimens used—CT, CCRT, or sequential CT and RT.^16^ Before accessing the data sets, researchers must sign an agreement to protect patient privacy. Researchers are allowed access to the CCHIA database for analyzing specific topics. Patient identification numbers in the data sets are encrypted, preventing specific patient identification.^17^ Here, the diagnoses of enrolled patients were confirmed according to pathological data, and patients with new HNSCC diagnoses had no other cancers or distant metastasis. The inclusion criteria were HNSCC (identified according to International Classification of Diseases, Ninth Revision, Clinical Modification [ICD-9-CM] codes 140.0–148.9), an age > 20 years, American Joint Committee on Cancer (AJCC) clinical cancer stage III or IV (locally advanced HNSCC without metastasis), and treatment by using surgery, CT, RT, CCRT, sequential CT and RT, or surgery with adjuvant treatment. The exclusion criteria were a history of cancer before diagnosis of HNSCC, distant metastasis, AJCC clinical cancer stage I or II, missing sex data, an age < 20 years, nasopharyngeal cancer, in situ carcinoma, sarcoma, and HNSCC recurrence. The index date was the date of the first diagnosis of HNSCC. In total, 21,174 patients with stage III or IV HNSCC were enrolled. To compare their outcomes, they were categorized into groups on the basis of treatment modality: Group 1 comprised those undergoing curative-intent aggressive treatments (namely curative surgery, surgery with adjuvant therapy, definitive RT, or CCRT [total irradiation dose ≥ 7000 cGy]); and Group 2 comprised those receiving BSC or palliative treatments including short-course and large-fraction RT (irradiation fraction size ≥ 250 cGy and total dose < 5000 cGy)^18–20^ or CT alone. Comorbidities were scored using the CCI.^11^ The examined comorbidities are listed in Supplemental Table 1. Only comorbidities observed 6 months before and after the index date were included. Comorbidities were determined and included according to the main ICD-9-CM diagnosis code for the first admission or >2 repeated main diagnosis codes for visits to the outpatient department. Age, sex, the CCI score, and the AJCC clinical cancer stage were controlled for or stratified in the analysis. The endpoint was the death rate among aggressive treatments, and Group 2 functioned as the control arm.

The cumulative incidence of death was estimated using the Kaplan–Meier method, and differences among treatment modalities were determined using the log-rank test. After adjustment for confounders, the Cox proportional method was used to model the time from the index date to all-cause and HNSCC-related death among patients undergoing the treatments. In multivariate analysis, hazard ratios (HRs) were adjusted for age, sex, the CCI score, and the clinical stage. Stratified analyses were performed to evaluate the mortality risk associated with aggressive and palliative or BSC treatment modalities and that associated with curative surgery or nonsurgical intervention among aggressive treatments for different CCI scores and ages. All analyses were performed using SAS Version 9.3 (SAS, Cary, NC). A 2-tailed *P* value < 0.05 was considered statistically significant.

## RESULTS

We enrolled 21,174 stage III or IV HNSCC patients without distant metastasis, with the median follow-up duration being 3.25 (interquartile range, 2.75) years. Group 1 (the aggressive treatment group) and Group 2 (the palliative or BSC treatment group) comprised 18,584 and 2590 patients, respectively (Table 1). In both groups, a higher proportion of male patients selected aggressive treatments (Group 1 vs Group 2: 17,450 [93.90%] vs 2338 [90.27%] patients), whereas a higher proportion of elderly patients with stage IV HNSCC selected palliative treatments or BSC (2104 [81.24%] vs 13,305 [71.59%] patients). The most predominant cancer site was the oral cavity (13,461 [72.43%] and 2041 [78.80%] patients in Groups 1 and 2, respectively).

**TABLE 1:**
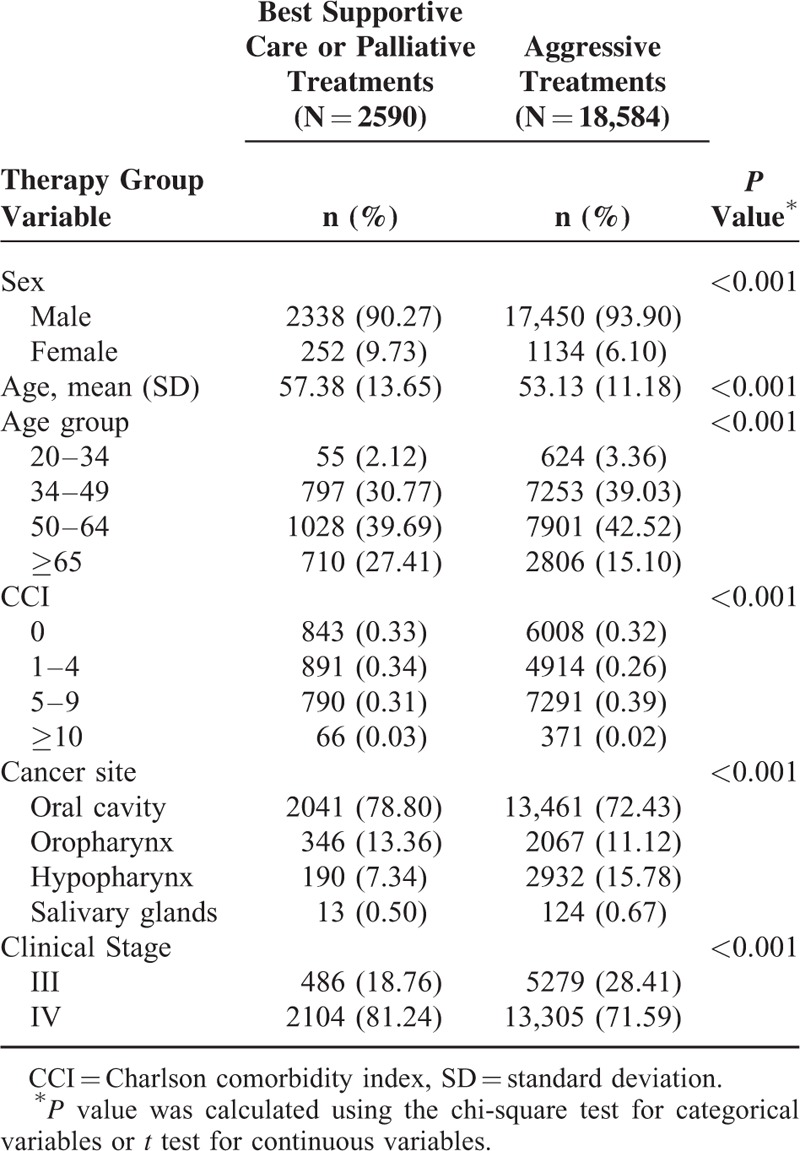
Characteristics of Patients With or Without Aggressive Treatments

**FIGURE 1:**
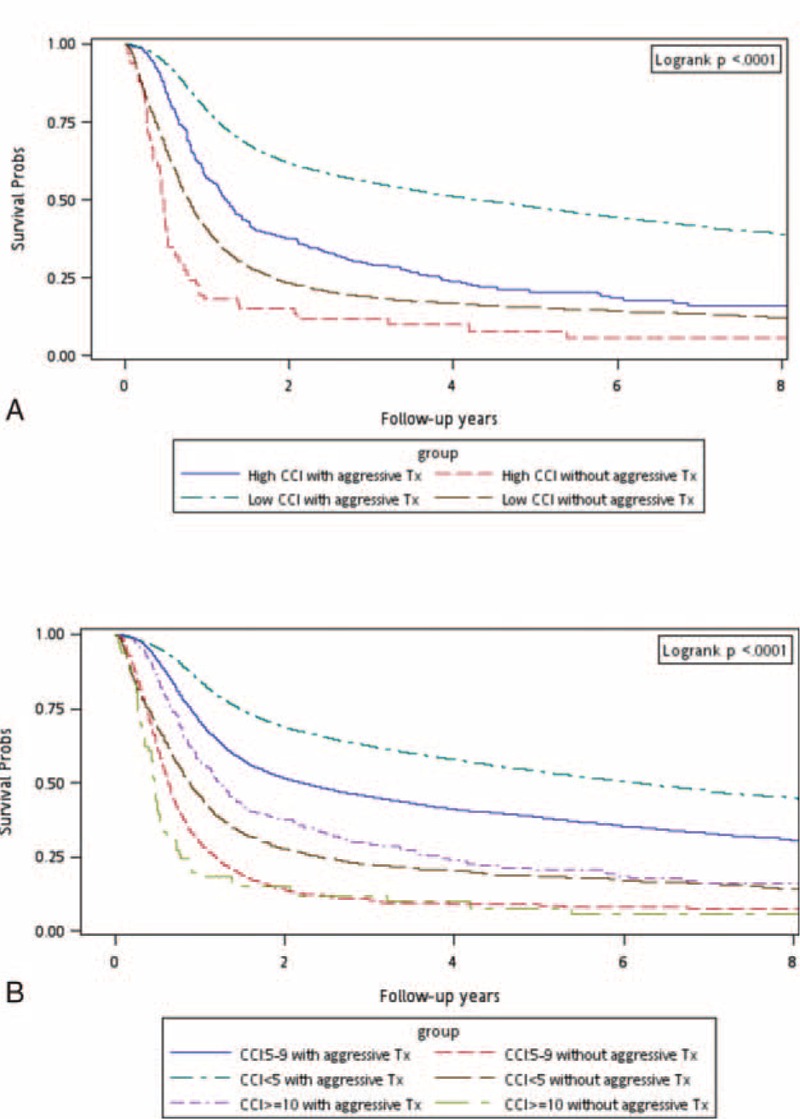
(A) Kaplan–Meier overall survival curves for head and neck cancer patients with different treatments and CCI scores (high CCI ≥ 10). (B) Kaplan–Meier overall survival curves for head and neck cancer patients with different treatments and CCI scores. CCI = Charlson comorbidity index.

**FIGURE 2:**
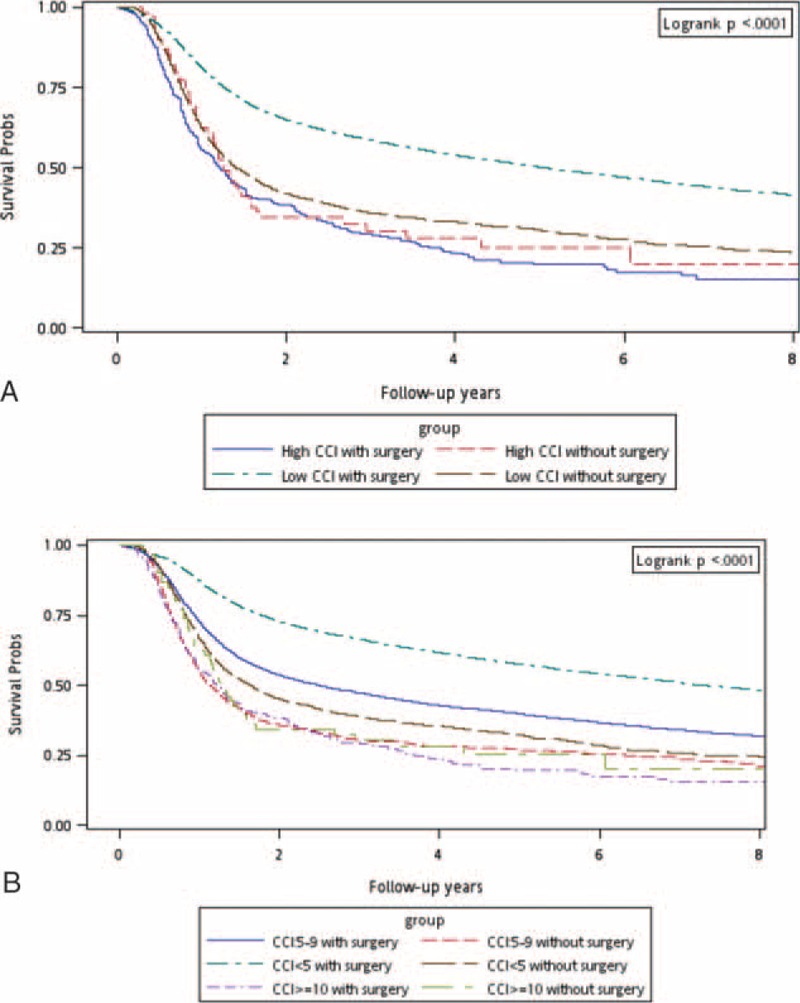
(A) Kaplan–Meier overall survival curves for head and neck cancer patients with aggressive treatments, with or without curative surgery, and with different CCI scores (high CCI ≥ 10). (B) Kaplan–Meier overall survival curves for head and neck cancer patients with aggressive treatment, with or without curative surgery, and with different CCI scores. CCI = Charlson comorbidity index.

A stratified analysis was performed to evaluate the mortality risk among treatment modalities for different CCI scores (<5, 5–9, and ≥10; Table 2). Group 2 functioned as the control arm for investigating the mortality risk after treatments. After adjustment for age, sex, and the clinical stage, adjusted hazard ratios (aHRs; 95% confidence intervals [CIs]) of overall death in Group 1 were 0.33 (0.31–0.35), 0.34 (0.31–0.36), and 0.37 (0.28–0.49) for CCI scores of <5, 5 to 9, and ≥10, respectively (Table 2). Another stratified analysis was performed to evaluate the mortality risk among treatment modalities for patients aged ≥65 and <65 years: among patients aged <65 years, aHRs (95% CIs) of overall death were 0.31 (0.29–0.33), 0.33 (0.30–0.36), and 0.28 (0.20–0.41) for CCI scores of <5, 5 to 9, and ≥10, respectively, whereas they were 0.42 (0.37–0.47), 0.40 (0.34–0.48), and 0.49 (0.30–0.80) for CCI scores of <5, 5 to 9, and ≥10, respectively, among those aged ≥65 years (Table 2). Figure 1A shows the overall survival curves of patients in the 2 treatment arms with high and low CCI scores (CCI ≥10 and <10, respectively). The highest overall survival rate was observed in Group 1 patients with low CCI scores (log-rank test, *P* < 0.0001). The 5-year overall survival rates were 50.37% and 24.53% for low and high CCI scores with aggressive treatments, respectively, whereas they were 17.55% and 9.09% for low and high CCI scores without aggressive treatments, respectively (Figure 1A). Figure 1B shows the overall survival curves of patients in the 2 treatment arms with CCI scores of <5, 5 to 9, and ≥10. The survival rates of Group 1 patients were higher than those of Group 2 patients with the same CCI scores (log-rank test, *P* < 0.0001): the 5-year overall survival rates were 56.95%, 40.52%, and 24.53% for CCI scores of <5, 5 to 9, and ≥10 with aggressive treatments, respectively, whereas they were 20.93%, 10.13%, and 9.09% for CCI scores of <5, 5 to 9, and ≥10 without aggressive treatments, respectively (Figure 1B). A stratified analysis was also performed to evaluate the mortality risk associated with curative surgery or curative nonsurgical intervention among the aggressive treatments for different CCI scores (<5, 5–9, and ≥10). Table 3 shows that curative surgical aggressive treatments were superior to nonsurgical aggressive treatments in patients with CCI scores of <5 or 5 to 9, but no improvement was observed in any patient with CCI scores of ≥10. The 30-day perioperative mortality in patients with CCI scores of ≥10 undergoing surgery was only 0.64%. However, complications after surgery were difficult to analyze. The aHRs (95% CIs) of all-cause death among patients undergoing curative surgical aggressive treatments were 1.13 (0.82–1.55), 0.67 (0.62–0.73), and 0.49 (0.46–0.53) for CCI scores of ≥10, 5 to 9, and <5, respectively. An analysis stratified according to age was performed to evaluate the mortality risk associated with curative surgery or nonsurgical intervention among the aggressive treatments for CCI scores of <5, 5 to 9, and ≥10. In HNSCC patients with CCI scores of <5 or 5 to 9, curative surgery improved overall survival, regardless of age, but no significant improvement was observed in overall survival in patients with high CCI scores (Table 3). Among patients with HNSCC aged <65 years undergoing curative surgery, the aHRs (95% CIs) of overall death were 0.46 (0.43–0.50), 0.66 (0.60–0.73), and 1.06 (0.74–1.52) for CCI scores of <5, 5 to 9, and ≥10, respectively, whereas they were 0.68 (0.59–0.78), 0.72 (0.58–0.90), and 1.20 (0.62–2.34) for CCI scores of <5, 5 to 9, and ≥10, respectively, among patients with HNSCC aged ≥65 years undergoing curative surgery. Figure 2A shows the overall survival curves of patients undergoing aggressive treatments with or without curative surgery and with high or low CCI scores. In Group 1, curative surgery resulted in higher overall survival in HNSCC patients with low CCI scores than in those with high CCI scores (log-rank test, *P* < 0.0001). The 5-year overall survival rates were 53.08% and 23.62% for low and high CCI scores with surgery, respectively, whereas for low and high CCI scores without surgery, they were 32.95% and 29.03%, respectively (Figure 2A). The 5-year overall survival rates were 60.71%, 42.03%, and 23.62% for CCI scores of <5, 5 to 9, and ≥10 with surgery, respectively, whereas for CCI scores of <5, 5 to 9, and ≥10 without surgery, they were 35.04%, 29.02%, and 29.03%, respectively (Figure 2B). However, in patients with high CCI scores, the curves of aggressive treatments with and without curative surgery overlapped. Figure 1B presents the overall survival curves of aggressive treatments with and without curative surgery for CCI scores of <5, 5 to 9, and ≥10. The results showed a higher survival rate in patients undergoing aggressive treatments with curative surgery who had CCI scores of <5 and 5 to 9 (log-rank test, *P* < 0.0001), and the curves of aggressive treatments with and without curative surgery overlapped in patients with CCI scores of ≥10.

**TABLE 2:**
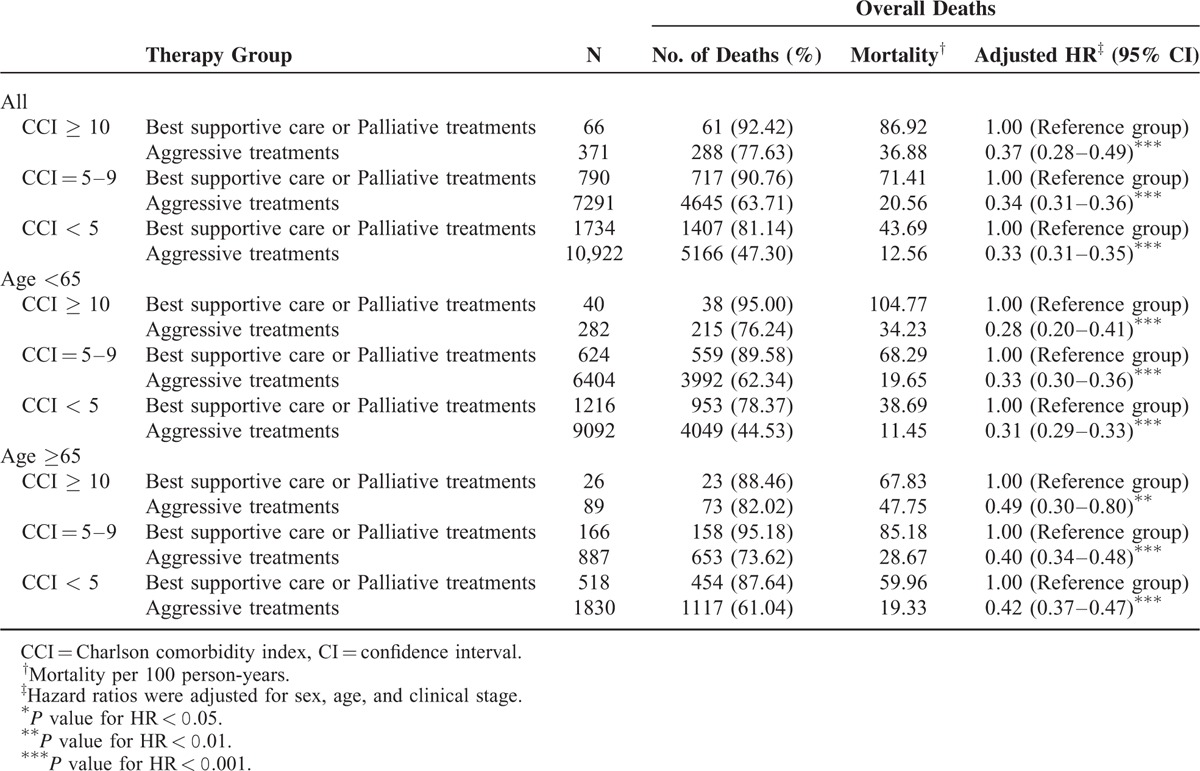
Effect of Aggressive Treatment on Overall Survival in Patients With Different CCI Scores

**TABLE 3:**
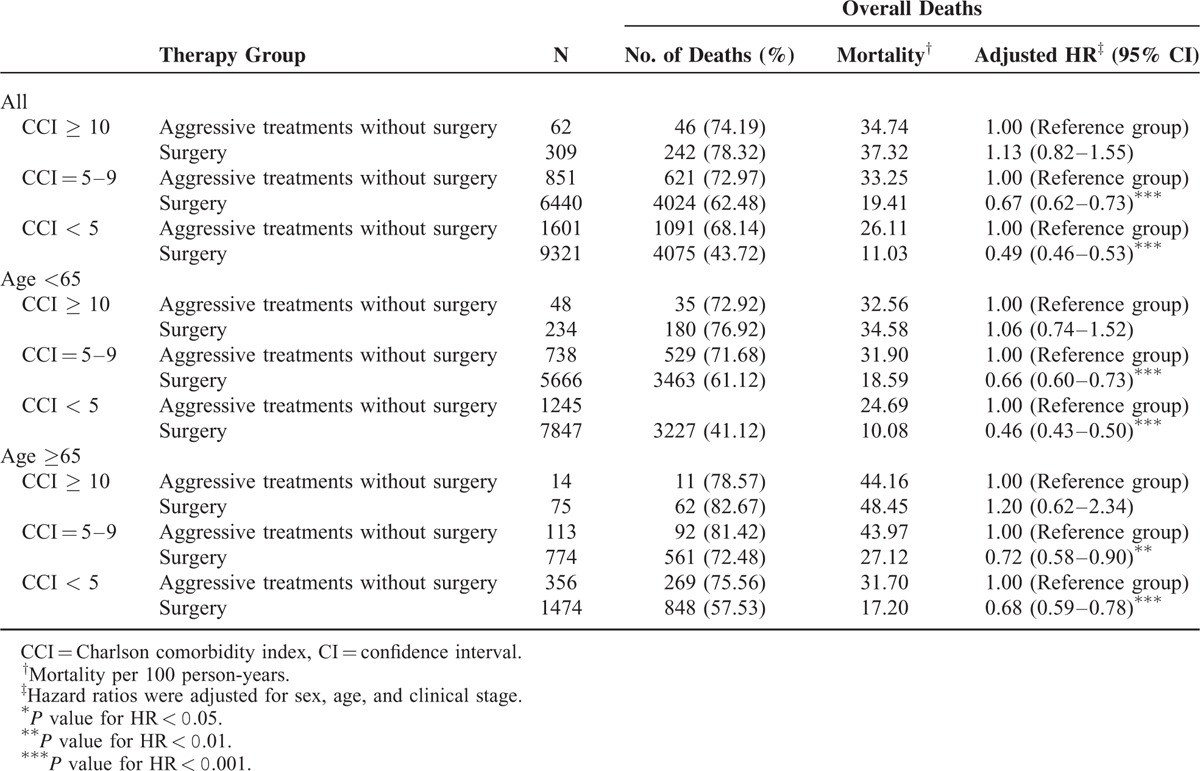
Effect of Surgery on Overall Survival and the Risk of Overall Death in Patients With Different CCI Scores

Among patients with high CCI scores, the 2 groups were statistically similar in sex, mean age, and clinical stage (Table 4). A higher proportion of Group 2 patients was aged ≥65 years (Group 1 vs Group 2: 26 [39.39%] vs 89 [23.99%] patients). Moreover, a higher proportion of Group 2 patients had cancers in the oral cavity and oropharynx.

**TABLE 4:**
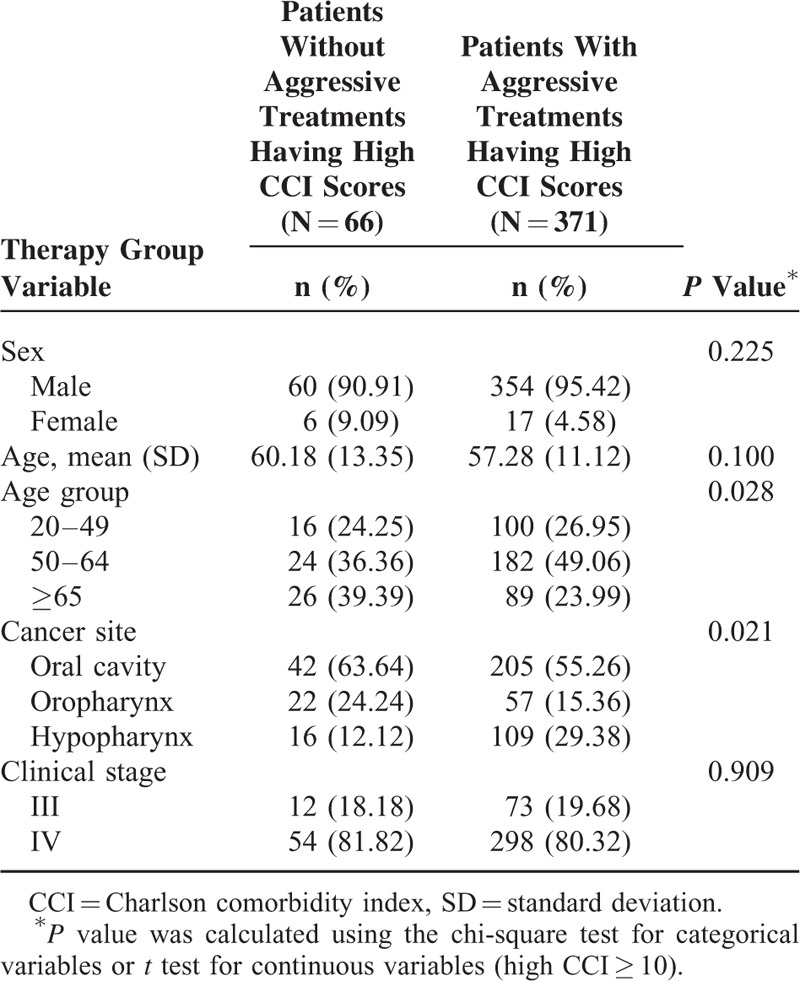
Characteristics of Patients With or Without Aggressive Treatments Having High CCI Scores

## DISCUSSION

The decisions regarding curative aggressive treatments for managing locoregionally advanced HNSCC are made by a multidisciplinary team including surgeons, medical oncologists, radiation oncologists and dentists, speech and swallowing pathologists, dieticians, and rehabilitation therapists, all with adequate expertise, and surgery is generally preferred in Taiwan because >70% of Taiwanese patients with HNSCC have oral cavity cancer^21,22^ and ∼80% of the patients chew betel nut.^22^ HNSCC, an aggressive cancer, has high locoregional recurrence and secondary primary cancer rates in Taiwan^22^; thus, postoperative RT with or without CT is strongly recommended.^3–5,23^ Definitive RT, CCRT, and sequential therapy are typically reserved for patients with medically inoperable unresectable or resectable cancers, where surgical resection cannot be accomplished with acceptable long-term functional consequences. The conventional treatment regimen includes administering a total radiation dose of 70 Gy over 7 weeks as a single fraction (2 Gy) once daily 5 days per week, with 50 to 60 Gy of radiation targeting elective areas; this treatment regimen is a feasible alternative to hyperfractionation or accelerated treatment approaches.^24^ However, multimodality therapies are often applied to HNSCC patients with considerably short survival. Elderly patients with HNSCC (herein, those aged ≥65 years) often have an impaired functional status that may hinder their ability to undergo and tolerate aggressive treatments. They are often excluded from clinical trials having defined standards of care, and they may experience high toxicity, particularly with treatment intensification.^25^ Therefore, the therapeutic decision for these patients can be considerably challenging. In our study, old age (≥65 years) did not seem to be a significant factor predicting poor overall survival for aggressive treatments (Table 2). These outcomes are consistent with a review by Van der Walde revealing that elderly and young patients have similar survival outcomes.^25^ Aggressive treatments were beneficial for HNSCC patients with high CCI scores, regardless of age; however, the aHRs tended to increase in elderly HNSCC patients relative to those of young patients with the same CCI scores (Table 2).

With aging of the population worldwide, the proportion of patients who are elderly at the time of HNSCC diagnosis will increase, contributing to increased aging-related health problems and comorbidities. Comorbidities are crucial not only for clinical decision-making but also for adjusting outcome data in retrospective analyses and for stratification in prospective studies. Furthermore, comorbidities are crucial prognostic factors for overall survival. Several tools can be used to evaluate comorbidity. The CCI^26^ is one of the most widely used tools for evaluating the effect of comorbidity on various cancers and noncancer conditions.^16,27–29^ Boje et al created a national cohort of 9388 patients with HNSCC diagnosed during 1992 to 2008 undergoing curative-intent RT from the DAHANCA database; data on comorbidity before HNSCC diagnosis were obtained from the National Patient Registry and adapted to the CCI. The authors reported that the performance of the CCI in stratifying patients according to overall survival was high.^15^ The authors also developed a revised head and neck comorbidity index (HN-CCI), and its performance in stratifying patients according to survival was also high; the CCI score was a strong independent prognostic factor for overall survival, with the HRs (95% CIs) being 1.16 (1.08–1.25), 1.34 (1.22–1.46), and 1.63 (1.51–1.80) for patients with mild, moderate, and severe CCI scores, respectively. Thus, the HN-CCI is highly recommended for assessing the comorbidity and prognostic staging of RT-treated patients with HNSCC.^14^ However, because the HN-CCI was designed only for RT-treated patients with HNSCC, its long-term validity requires further evaluation.

Comorbidities are common among patients with HNSCC and have negative prognostic effects on overall survival.^14,15^ In our study, aggressive treatments, namely curative surgery, surgery with adjuvant therapy, definitive RT, and CCRT (total irradiation dose ≥7000 cGy), increased overall survival in patients with HNSCC, regardless of age, and CCI score (Table 2; Figure 1). Nevertheless, the increasing aHR trend showed that survival decreased as CCI scores increased; this trend was similar to that reported previously.^14,15^ Similarly, survival decreased with increasing age (Table 2). Figure 1 shows the overall survival curves of the 2 treatment arms for CCI scores of <5, 5 to 9, and ≥10. In patients with the same CCI score, aggressive treatments resulted in a higher survival rate compared with that of BSC or palliative treatments (log-rank test, *P* < 0.0001). In previous studies, cancer-specific death was not affected by comorbidities, suggesting that comorbidities, rather than cancer, cause the death;^14,15^ these findings are similar to our outcomes. Our data also showed that aggressive treatments do not reduce cancer-specific death in HNSCC patients with high CCI scores (data not shown).

To the best of our knowledge, this is the first study demonstrating that aggressive treatments can improve survival even in elderly and critically ill patients with HNSCC (aHR, 0.49; 95% CI, 0.30–0.80) by using stratified analyses evaluating the mortality risk among treatment modalities for different CCI scores and ages.

A randomized controlled trial compared aggressive treatments with and without surgery and showed that surgery is a superior treatment for advanced HNSCC (stage III or IV nonmetastatic HNSCC);^30^ however, the trial included no elderly patients or patients with multiple comorbidities, and thus, the suitability of curative surgery for these patients remained unclear. Our study showed no significant differences between the overall survival rates of elderly (>65 years) and younger HNSCC patients with high CCI scores after surgery (Table 3) and demonstrated a consistent survival trend among elderly and young HNSCC patients with the same CCI scores. Therefore, extended surgical treatments should be offered to both elderly and young patients with similar comorbidities. These results are consistent with those of Sesterhenn and Peters, who demonstrated that advanced age may not be a contraindication for major head and neck surgery.^31,32^ However, Table 3 shows that curative surgical aggressive treatments were superior to nonsurgical aggressive treatments in HNSCC patients with low CCI scores, but no improvements were observed in those with high CCI scores. Furthermore, for the first time, our analyses of the mortality risk associated with curative surgery or nonsurgical intervention among aggressive treatments modalities for HNSCC patients with high or low CCI scores stratified according to age indicated that surgery does not improve the survival rate in HNSCC patients with high CCI scores, regardless of age. In addition, many patients in the nonsurgical aggressive treatment group had medically inoperable or unresectable HNSCC, implying that Group 1 patients had more advanced HNSCC. The harm caused by curative surgery (aHR, 1.13) in HNSCC patients with high CCI scores may have been underestimated. Although age did not significantly affect overall survival in patients with HNSCC, we adjusted the analysis for age, regardless of whether surgery was performed. However, the highest aHR (95% CI) of overall death was 1.20 (0.62–2.34) in patients with HNSCC aged ≥65 years and in those with high CCI scores. Our results for HNSCC patients with low CCI scores are consistent with those of the randomized controlled trial by Soo, which indicated that surgery results in the optimal overall survival.^30^

In clinical practice, aggressive treatments are extremely beneficial for patients with HNSCC, even in elderly patients and those with high CCI scores. Aggressive treatments reduced overall death by >60% compared with BSC or palliative treatments in patients with the same CCI scores. Even in elderly patients with HNSCC and those with high CCI scores, aggressive treatments reduced overall death by 51% (Table 2). Curative surgery was beneficial for HNSCC patients with low CCI scores and was necessary for HNSCC patients with high CCI scores. However, surgical aggressive treatments did not improve overall survival in HNSCC patients with high CCI scores (Table 3). Nevertheless, we recommend that elderly patients with HNSCC and those with multiple comorbidities undergo aggressive treatments rather than BSC or palliative treatments. Nonsurgical aggressive treatments including definitive RT or CCRT (total irradiation dose ≥ 7000 cGy) might be more suitable for HNSCC patients with high CCI scores (Table 3).

The strength of this study is the large sample size. The results suggested that aggressive treatments can reduce the incidence of death in patients with HNSCC. This was also the first study indicating the optimal therapeutic decisions for patients with HNSCC according to age and comorbidity; aggressive treatments are a more appropriate treatment decision, and this should be considered in future clinical studies. However, this study has limitations. First, the toxicity induced by curative-intent aggressive treatments could not be determined; therefore, the treatment-related mortality estimates may have been biased. Second, information regarding the human papillomavirus (HPV) test is not recorded in the databases used in this study; hence, the effect of different treatments on HPV-positive or -negative patients could not be examined. Third, all investigated patients with HNSCC were from an Asian population, and racial susceptibility was unclear; hence, our results should be cautiously extrapolated to non-Asian populations. Fourth, the relatively small number of patients with high CCI scores might limit the generalizability of the conclusions in this study; a large-scale randomized trial, in which carefully selected patients undergoing suitable aggressive treatments and palliative or supportive care approaches are used for comparison, is essential to obtain crucial information regarding population specificity and disease occurrence. Fifth, diagnoses of all comorbid conditions were completely dependent on ICD-9-CM codes; nevertheless, the Taiwan NHI Administration randomly reviews charts and interviews patients to verify the accuracy of the diagnoses, and hospitals with outlier chargers or practices may undergo an audit and subsequently receive heavy penalties if malpractice or discrepancies are identified. Finally, the database contains no information on tobacco use, alcohol consumption, dietary habits, socioeconomic status, or body mass index, all of which may be mortality risk factors. Nevertheless, given the magnitude and statistical significance of the observed effects in this study, these limitations are unlikely to have affect the conclusions.

## CONCLUSIONS

Aggressive treatments can be beneficial even for critically ill and elderly patients with HNSCC, and they reduced overall death by >60% compared with BSC or palliative treatments in patients with the same CCI score. In elderly patients with HNSCC and those with high CCI scores, aggressive treatments reduced overall death by 51%. Nonsurgical aggressive treatments including definitive RT and CCRT (total irradiation dose ≥7000 cGy) might be suitable for HNSCC patients with high CCI scores.

## Supplementary Material

Supplemental Digital Content
