# Supplementary material for: Curative-Intent Aggressive Treatment Improves Survival in Elderly Patients With Locally Advanced Head and Neck Squamous Cell Carcinoma and High Comorbidity Index
Source: Medicine (Baltimore). 2016 Apr 8;95(14):e3268. doi: 10.1097/MD.0000000000003268 (PMC4998798; doi:10.1097/MD.0000000000003268)
Supplement: Supplemental Digital Content [file medi-95-e3268-s001.docx]

| **Supplementary Table 1.** Prevalence and score of diseases within Charlson comorbidities index among HNSCC patients in different age groups | | | | | | | | | |
| --- | --- | --- | --- | --- | --- | --- | --- | --- | --- |
| Therapy Group | Total  (N=21174) | | Age:20-49  (N=8729) | | Age:50-64  (N=8929) | | Age:65+  (N=3516) | | Charlson Score |
| Variable | n | (%) | n | (%) | n | (%) | n | (%) |  |
| Myocardial infarction | 148 | (0.70) | 22 | (0.25) | 73 | (0.82) | 53 | (1.51) | 1 |
| Congestive heart failure | 477 | (2.25) | 62 | (0.71) | 189 | (2.12) | 226 | (6.43) | 1 |
| Peripheral vascular disease | 157 | (0.74) | 22 | (0.25) | 62 | (0.69) | 73 | (2.08) | 1 |
| Cerebrovascular disease | 1110 | (5.24) | 132 | (1.51) | 490 | (5.49) | 488 | (13.88) | 1 |
| Dementia | 134 | (0.63) | 4 | (0.05) | 11 | (0.12) | 119 | (3.38) | 1 |
| Chronic pulmonary disease | 1723 | (8.14) | 316 | (3.62) | 683 | (7.65) | 724 | (20.59) | 1 |
| Rheumatic disease | 124 | (0.59) | 31 | (0.36) | 50 | (0.56) | 43 | (1.22) | 1 |
| Peptic ulcer disease | 2359 | (11.14) | 726 | (8.32) | 1069 | (11.97) | 564 | (16.04) | 1 |
| Mild liver disease | 2944 | (13.90) | 1326 | (15.19) | 1260 | (14.11) | 358 | (10.18) | 1 |
| Diabetes without chronic complication | 3450 | (16.29) | 814 | (9.33) | 1761 | (19.72) | 875 | (24.89) | 1 |
| Diabetes with chronic complication | 587 | (2.77) | 103 | (1.18) | 330 | (3.70) | 154 | (4.38) | 2 |
| Hemiplegia or paraplegia | 106 | (0.50) | 18 | (0.21) | 47 | (0.53) | 41 | (1.17) | 2 |
| Renal disease | 473 | (2.23) | 78 | (0.89) | 217 | (2.43) | 178 | (5.06) | 2 |
| Any malignancy, | 2372 | (11.20) | 852 | (9.76) | 1058 | (11.85) | 462 | (13.14) | 2 |
| Moderate or severe liver disease | 238 | (1.12) | 110 | (1.26) | 104 | (1.16) | 24 | (0.68) | 3 |
| Metastatic solid tumor | 8126 | (38.38) | 3651 | (41.83) | 3434 | (38.46) | 1041 | (29.61) | 6 |
| AIDS/HIV | 22 | (0.10) | 14 | (0.16) | 8 | (0.09) | 0 | (0.00) | 6 |
|  | | | | | | | | | |
